# Third Set of Teeth

**Published:** 1897-08

**Authors:** S. B. Palmer

**Affiliations:** Syracuse, N. Y.


### THIRD SET OF TEETH.

BY S. B. PALMER, M.D.S., SYRACUSE, N. Y.

An extract from an article in the June, 1896, issue of the International Dental Journal reads as follows:

"The Sunday *Herald* of Syracuse, New York, reports a case at considerable length of one James Slattery, of that city, who has apparently erupted late in life a portion of the full denture, with the prospect of more teeth presenting in the near future. Dr. S. B. Palmer, of Syracuse, made an examination of the case. . . .

"This seems the best attested report of third dentition we have met with on record, and it is hoped Dr. Palmer will secure testimony as to the character of the set prior to becoming edentulous through advancing years. The dental profession has regarded a third set of teeth as a myth, and with some reason, as most of the so-called third sets have proved, on investigation, to be simply delayed dentition of the regular second set."

The history of this case, together with facts which came under my own observation, and statements from Mr. Slattery himself, also from an unmarried daughter who had her home with him, I believe establishes a case of third dentition beyond a doubt.

James Slattery was born in Ireland in 1805. In 1832 he came to Napanee, Ontario, and two years later settled in Liverpool, near Syracuse, New York. He soon became a prominent salt manufacturer, owning his own plant. With the decadence of that once lucrative industry he, in 1867, removed to his farm and devoted himself to market-gardening. Mr. Slattery was a remarkably well-preserved man, and enjoyed robust health. When in his prime he was a well-developed man of six feet two inches, weighing two hundred and thirty pounds.

On mentioning the object of my call, I learned that he had carefully avoided notoriety, and had concealed the fact from his family as long as possible for fear that people would joke him about it, and would say that "Slattery was a baby and was just cutting his teeth." A moment's examination was convincing that the case was not a myth, and great care was taken in obtaining notes from which this report is a summary.

In the inferior maxilla were eight teeth located as follows: four incisors and two cuspidati, and two bicuspids on the right side. The cuspid on the left side and the second bicuspid on the right side were somewhat loose. The other teeth were firm, of usual size and length. They were somewhat overlapped from crowding. The color suited the age, which I attributed to smoking. The teeth showed no wear, as teeth with the use of seventy-five or eighty years would have done, in holding a pipe. He showed how he could do it by placing his pipe in his mouth and pressing it against his gums above. He said he could not do that for twenty years owing to the loss of his teeth. On the left side, in place of the two posterior teeth ordinarily found, were three parts of teeth resembling roots, having grown up partially out of the gums. They were not firm or well-developed teeth. The man insisted that they also appeared within the time mentioned, two years previously, and that they belonged to the same set. I am satisfied that they were roots belonging to the second set, which had been covered by the gums or had escaped his notice until attention was called by the eruption of the teeth already mentioned. The other portions of the jaw showed no signs of further eruption of teeth, as considerable absorption had taken place.

An examination of the superior maxilla plainly showed that the third set, more numerous than those in the inferior, would soon make their appearance. The jaw was thick and full, nearly all the way back raising the lip and cheeks, giving the fulness peculiar to a child's jaw of six or seven years, or about the period of the eruption of the teeth of the permanent set. Mr. Slattery said his "jaws pained him very much; that the new teeth pricked the gums like needles," and "there will be a full set, except where I had a tooth pulled. I never had but one pulled, and a piece of bone came out with it. Now there isn't any tooth coming in there at all; that's the only vacancy, and that aint sore." I examined the "vacancy;" the depression was unusually deep owing to the growth of new process to support the incoming teeth. This closed the examination, and I intended to make another call in ten or twelve months.

Mr. Slattery died suddenly of disease of the heart, April 24, 1896, a little over eight months from the examination. I did not learn of his death at the time, but made two calls upon the family, from which I gained the following information: Having requested his daughter to observe developments, I believe her statements correct. The superior maxilla became painful, and the teeth erupted nearly at the same time with wonderful rapidity, so that before his death the upper denture was complete, with the exception of the place where the tooth was extracted. I asked as to the color of the teeth. She said they were yellow, much like the teeth of an old person. They were well developed. Soreness had abated and mastication was restored. I made inquiries respecting the teeth reported loose in the inferior maxilla. "They became firm like the others."

The *cause* of the crumbling away of the second set without severe pain, and that none were extracted save the one already mentioned, Miss Slattery could give no information.

That a man of robust health, and otherwise so well-preserved, should lose his teeth, and roots as well, calls for some constitutional change not usually met with. It could not be charged to pyorrhœa, because none of the teeth came out sound.

A patient whose teeth have been under my care for fourteen years or more are fast going in a like manner, giving little or no pain. He is a plumber, and has been for years engaged on soldering roofing and galvanized iron cornices, etc. He said the breathing of the fumes from muriate of zinc (soldering fluid) had destroyed his teeth. The case before us seems to bear upon the same principle, and it is reasonable to suppose that the manner in which the roots came from the maxilla prepared the way for the conditions which followed.

Mr. Slattery was a salt manufacturer. For many years much of his labor was done in a "salt block." Evaporation was by boiling, and the warm vapor at times almost blinding. When not engaged in dipping salt from the boiling-kettles, the packing of moist salt was performed in an atmosphere highly charged with chloride of sodium.

The suggestions as to the cause of the loss of the teeth, etc., are my own. The history of the case as obtained from the family, with my personal observations, convinces me that nature gave to Mr. Slattery a third set of teeth as above described.

